# Effect of Bonding Height on Force‐Moment Generation in Orthodontic Fixed Lingual Retainers: An In Vitro Study

**DOI:** 10.1002/cre2.70396

**Published:** 2026-06-28

**Authors:** Francesca Thaden, Linus Hötzel, Benedikt Dotzer, Matthias Mertmann, Andrea Wichelhaus, Hisham Sabbagh

**Affiliations:** ^1^ Department of Orthodontics and Dentofacial Orthopedics LMU University Hospital, LMU Munich Munich Germany

**Keywords:** adverse effects, biomechanics, force, orthodontic retainer, torque

## Abstract

**Objectives:**

To investigate the effect of different bonding heights on force‐moment generation in orthodontic fixed lingual retainers under loading in a biomechanical setup.

**Materials and Methods:**

Three commercially available lingual retainers were evaluated: two conventionally fabricated stainless‐steel retainers (R1, R2) and one CAD/CAM‐manufactured nickel‐titanium retainer (R3). Using a biomechanical test setup, vertical forces and labiolingual moments were recorded and applied in three dimensions in response to a standardized vertical displacement of 0.3 mm. The effect of three standardized bonding heights (incisal, middle, and gingival thirds of the clinical crown) was assessed.

**Results:**

Both forces and moments increased with vertical loading. Statistically significant differences were observed among retainer materials, whereas bonding height did not significantly affect the outcomes. At 0.3 mm vertical displacement, the lowest force and moment were recorded for R1 bonded incisally (0.59 ± 0.37 N; 3.48 ± 0.37 Nmm), and the highest for R3 at mid‐crown bonding height (1.40 ± 0.13 N; 8.70 ± 1.19 Nmm).

**Conclusions:**

Retainer type was found to have a greater influence on force and moment development than bonding height. Multistranded stainless‐steel retainers may be more susceptible to deformation under occlusal loading. Further studies are required to determine the clinical relevance of bonding height in fixed lingual retainers.

## 1 Introduction

Following active orthodontic treatment, retention is required to prevent relapse and to maintain posttreatment stability (Fleming and Pandis 2024). Both removable and fixed retainers are suitable and widely used for that purpose (Martin 2023). However, long‐term adherence with removable retainers commonly declines over time, with most patients discontinuing wear within a few years (Krämer et al. 2023). Fixed lingual retainers are therefore frequently used for long‐term retention but have become associated with complications known as inadvertent tooth movements (ITMs) (Katsaros et al. 2007). ITMs can present as a torque difference between two neighboring incisors (“X‐Effect”) or as a marked labial or lingual torquing of one or both canines (“Twist‐Effect”) (Katsaros et al. 2007). While milder cases have been shown to correct spontaneously after the removal of the fixed retainer (Katsaros et al. 2007), more severe cases can require periodontal surgery, orthodontic retreatment (Shaughnessy et al. 2016), or even extraction of the affected tooth (Singh 2021). ITMs have been extensively researched, yet studies evaluating patient‐related factors (Klaus et al. 2024, 2020; Kučera and Marek 2016; Wolf et al. 2016) and the influence of different retainer materials (Arnold et al. 2016; Sifakakis et al. 2015, 2011) have not conclusively determined their etiology. Some studies postulated that retainer bonding height (RBH) might influence ITMs, but only a few studies have investigated this aspect. A study by Jahanbin et al. (2014) used the finite element method (FEM) and reported that a more incisal bonding height was more effective in maintaining stability after simulated vertical loading. Another in vitro study similarly compared two RBHs and found that greater forces were required to displace teeth bonded to the more incisally placed retainer (Ohtonen et al. 2021). Notably, the influence of RBH on multistranded retainers, which have been most frequently associated with ITM development, has not yet been evaluated to the authors’ knowledge, despite these being among the most commonly used retainer types (Padmos et al. 2018). The aim of this study was to investigate the effect of three standardized RBHs on force‐moment generation in different orthodontic fixed lingual retainers. The null hypothesis was that no significant differences in force or moment development would be observed between retainer types or among different bonding heights.

## 2 Materials and Methods

The biomechanical test stand FRANS (Force/Torque Analysis System) was used to investigate force and moment development in the fixed lingual retainers under vertical loading (Thaden et al. 2024). The setup consists of a measurement table with tooth sockets, a micrometer‐driven vertical displacement unit, and a six‐axis force‐torque sensor (resolution: force 0.0125 N; moment 0.626 Nmm) to record forces and moments at the retainer. A mandibular model including teeth 33–43 was used for the experiments. Two stainless‐steel (SS) retainers and one nickel‐titanium (NiTi) retainer were compared (Table 1).

**Table 1:** Characteristics of the investigated lingual fixed retainers. The material, cross‐sectional dimensions, shape, and manufacturing methods are listed.

Retainer	Material	Dimension	Shape	Manufacturing
R1	SS	0.0215”	Stranded, round	Bent using pliers
R2	SS	0.010” × 0.029”	Stranded, rectangular, flat	Bent by hand
R3	NiTi	0.014” × 0.014”	Rectangular	CAD/CAM manufactured; laser cut from a sheet

Abbreviations: CAD/CAM, computer‐aided‐design and computer‐aided‐manufacturing; NiTi, nickel‐titanium; SS, stainless‐steel.

**Figure 1:**
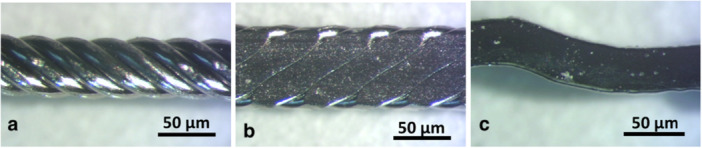
Microscope images of the analyzed retainers: (a) Stranded round retainer R1, (b) stranded rectangular flat retainer R2, and (c) laser‐cut retainer R3.

Retainer R1 was stranded, round, and manually bent using pliers (Figure 1a). Retainer R2 was stranded, flat, and hand‐bent (Figure 1b). Retainer R3 was manufactured using computer‐aided‐design and computer‐aided‐manufacturing (CAD‐CAM) and produced by laser‐cutting a NiTi sheet (Figure 1c).

**Figure 2:**
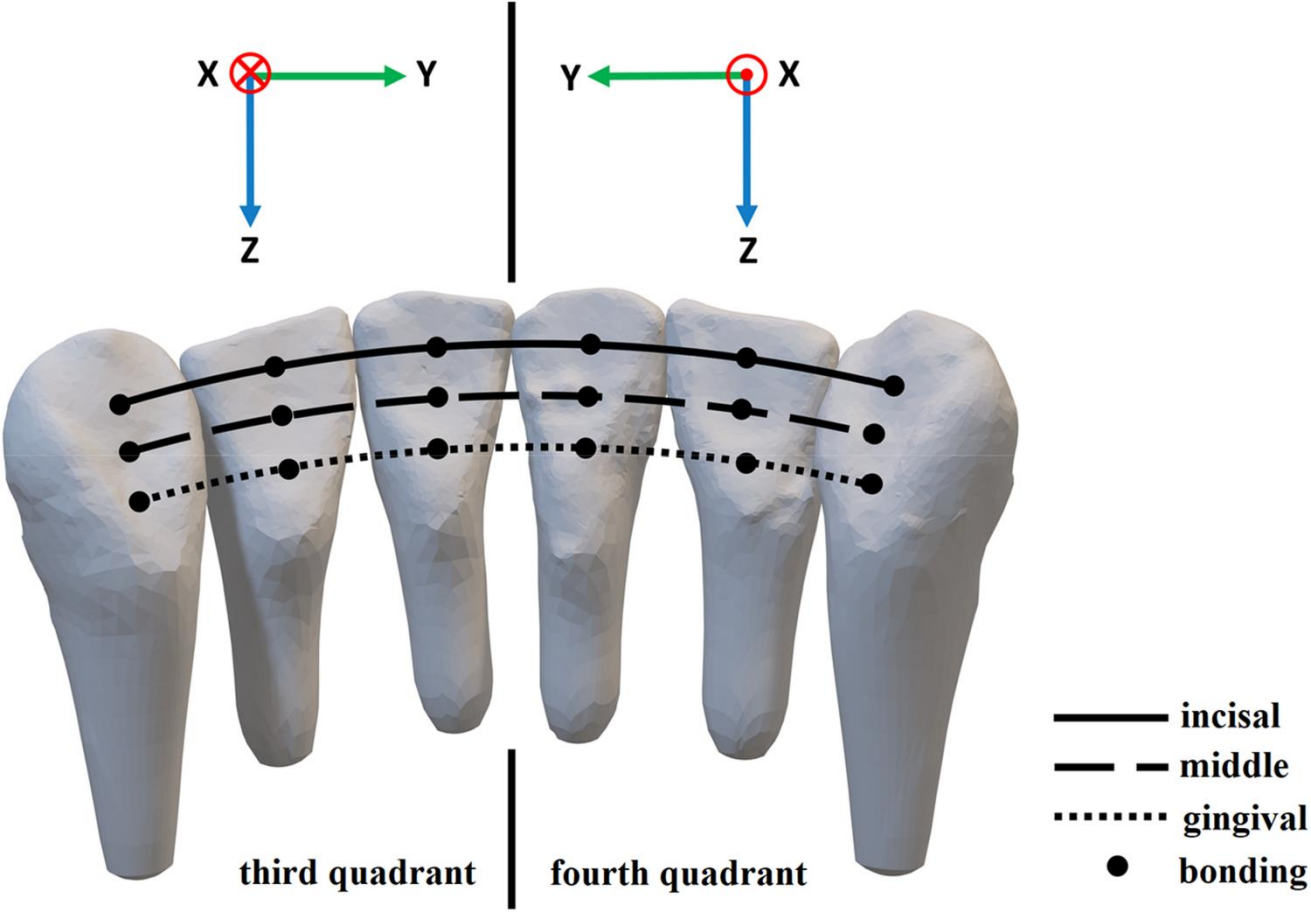
Retainer bonding height (RBH): Incisal bonding height (continuous line), middle bonding height (discontinuous line), and gingival bonding height (dotted line). Larger dots indicate the position of the bonding site.

Three standardized RBHs were defined (Figure 2). RBH standardization was achieved by using a marked reference model during manual bending of retainer groups R1 and R2. For the retainer group R3, the distinct RBHs were digitally implemented by the manufacturer. The retainers were bonded in the incisal, middle, and gingival third of the crown in equal numbers (*n* = 5 per subgroup, total measurement samples = 45).

The experimental models were fabricated using a 3D printer with a z‐resolution of 100 µm (Form 3B+, Grey Resin V4, Formlabs Inc., Somerville, MA, USA) and post‐processed according to the manufacturer's protocol. Each model extended from canine to canine and included cylindrical base structures to simulate tooth roots. After verifying passive retainer fit on the reference model, silicone transfer guides were fabricated, and the retainers were bonded to the experimental models using flowable composite (Transbond LV, 3M, St. Paul, MN, USA). After bonding of the retainers, a fine cutting disc was used to separate the teeth, thereby isolating them biomechanically.

**Figure 3:**
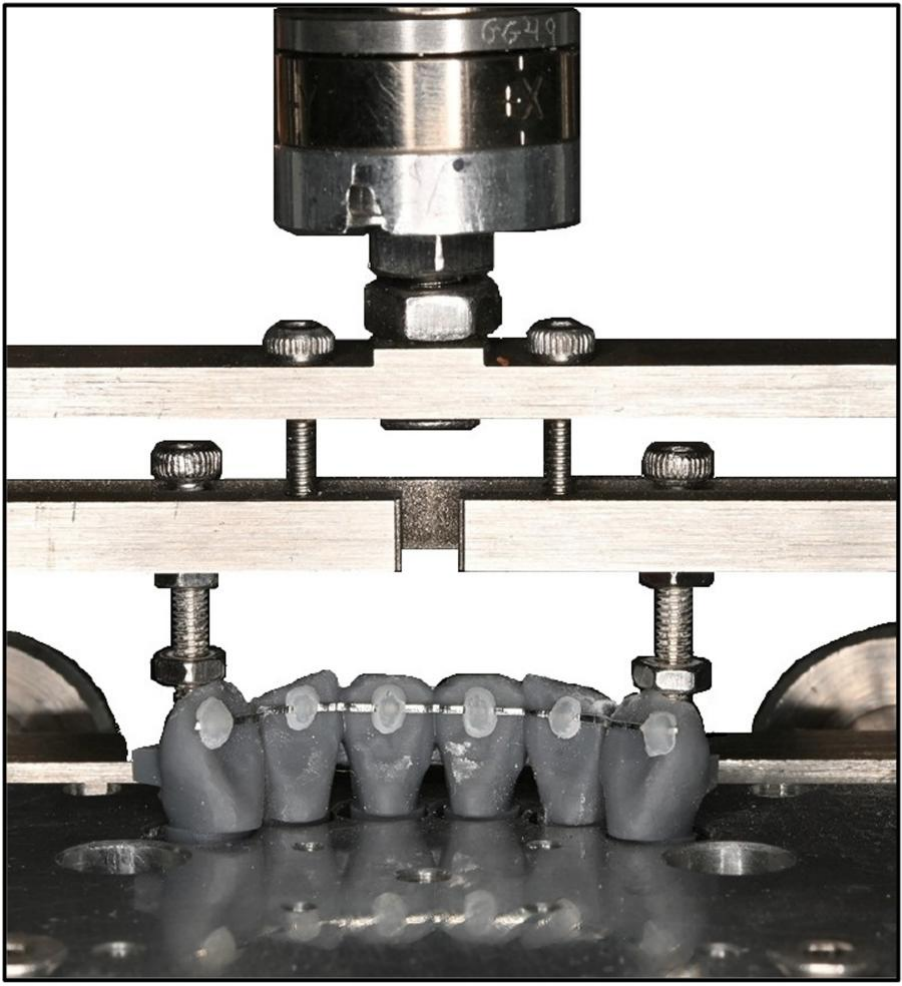
The FRANS measuring system, magnified to show the base plate and experimental model, as well as the sensor. The reference teeth, the canines, are connected to the sensor via the M2 screws and the metal connector piece. In this instance, an R3 retainer (CAD/CAM manufactured NiTi) with an incisal RBH is shown.

The experimental models were mounted into the FRANS measurement table (Figure 3) and connected to the displacement unit with M2 screws. The experimental temperature was maintained at 36.0 ± 1°C using a fan heater (PiccoVent, RO/SE Blechverarbeitung GmbH & Co. KG, Bad Birnbach, Germany).

Before each measurement was initiated, the system was digitally calibrated and the forces and moments were set to zero. Loading was applied by actuating the micrometer screw gauge, producing a continuous vertical displacement of the model at a crosshead speed of 0.5 mm/min. Data acquisition was terminated at a vertical displacement of 0.3 mm. Each retainer was tested once to avoid material fatigue. All measurements were performed by a single operator (FT) to minimize inter‐operator variability. Specimens exhibiting failures, such as debonding, were excluded from the final analysis. Data were collected using LabVIEW 2020 (NI, Austin, TX, USA) and processed in Excel 2016 (Microsoft Corporation, Redmond, WA, USA) and OriginPro 2022b (OriginLab Corporation, Northampton, MA, USA). Statistical analyses were performed with IBM SPSS Statistics 27 (IBM Corp., Armonk, NY, USA). Normality was assessed using the Shapiro–Wilk test. Group comparisons were conducted using the Kruskal–Wallis test with Bonferroni correction.

## 3 Results

The following analysis focuses on the vertical force (*F*_z_), which directly reflects the applied vertical displacement, and the labiolingual moment (*M*_y_), as this is the plane in which ITMs are observed clinically. *F*_z_ and *M*_y_ were recorded continuously, and values at displacements of 0.1, 0.2, and 0.3 mm are summarized in Table 2.

**Table 2:** Development of vertical force (*F*_Z_) and labiolingual moment (*M*_Y_) in retainers bonded at an incisal, middle, and gingival RBH.

Retainer	Displacement [mm]	Incisal bonding height		Middle bonding height		Gingival bonding height	
		*F*_z_ (SD) [N]	*M*_y_ (SD) [Nmm]	*F*_z_ (SD) [N]	*M*_y_ (SD) [Nmm]	*F*_z_ (SD) [N]	*M*_y_ (SD) [Nmm]
R1	0.1	0.28 (0.14)	1.70 (0.85)	0.35 (0.04)	2.16 (1.59)	0.33 (0.09)	1.86 (0.46)
	0.2	0.43 (0.26)	2.57 (1.59)	0.61 (0.45)	3.70 (0.42)	0.50 (0.12)	2.79 (0.64)
	0.3	0.59 (0.37)	3.48 (2.23)	0.88 (0.63)	5.30 (4.04)	0.76 (0.25)	4.35 (1.58)
R2	0.1	0.46 (0.41)	2.70 (2.48)	0.42 (0.22)	2.41 (1.34)	0.82 (0.42)	4.71 (2.69)
	0.2	0.99 (0.70)	5.80 (4.48)	0.84 (0.48)	5.02 (3.09)	1.03 (0.36)	5.67 (1.58)
	0.3	1.29 (0.70)	7.31 (4.15)	1.22 (0.80)	7.13 (4.57)	1.06 (0.30)	5.70 (1.30)
R3	0.1	0.55 (0.17)	3.66 (1.14)	0.55 (0.05)	3.41 (0.42)	0.53 (0.20)	3.80 (1.31)
	0.2	0.85 (0.19)	5.28 (0.98)	0.97 (0.09)	5.82 (0.82)	0.88 (0.28)	6.33 (2.09)
	0.3	1.13 (0.25)	6.88 (1.14)	1.40 (0.13)	8.70 (1.19)	1.14 (0.25)	8.53 (2.41)

Of the initial 45 samples, 4 were excluded due to bonding failures. No retainer fractures were observed. Among the retainer types, R1 consistently showed the lowest force and moment development across all RBHs (incisal: 0.59 ± 0.37 N and 3.48 ± 2.23 Nmm; middle: 0.88 ± 0.63 N and 5.30 ± 4.04 Nmm; gingival: 0.76 ± 0.25 N and 4.35 ± 1.58 Nmm). The highest values were recorded for R2 at the incisal RBH (1.29 ± 0.70 N and 7.31 ± 4.15 Nmm) and for R3 at the middle (1.40 ± 0.13 N and 8.70 ± 1.19 Nmm) and gingival (1.14 ± 0.25 N and 8.53 ± 2.41 Nmm) RBHs. Comparing RBH, retainers R1 and R3 showed the lowest forces and moments at the incisal RBH (R1: 0.59 ± 0.37 N and 3.48 ± 2.23Nmm, R3: 1.13 ± 0.25 N and 6.88 ± 1.14Nmm), and at the gingival RBH for R2 (1.06 ± 0.30 N and 5.70 ± 1.30Nmm). In the R1 and R2 groups, standard deviations were higher than in R3 and increased over the course of loading, whereas they remained relatively constant in R3. Overall, variability was greater for *M*_y_ than for *F*_z_.

**Figure 4:**
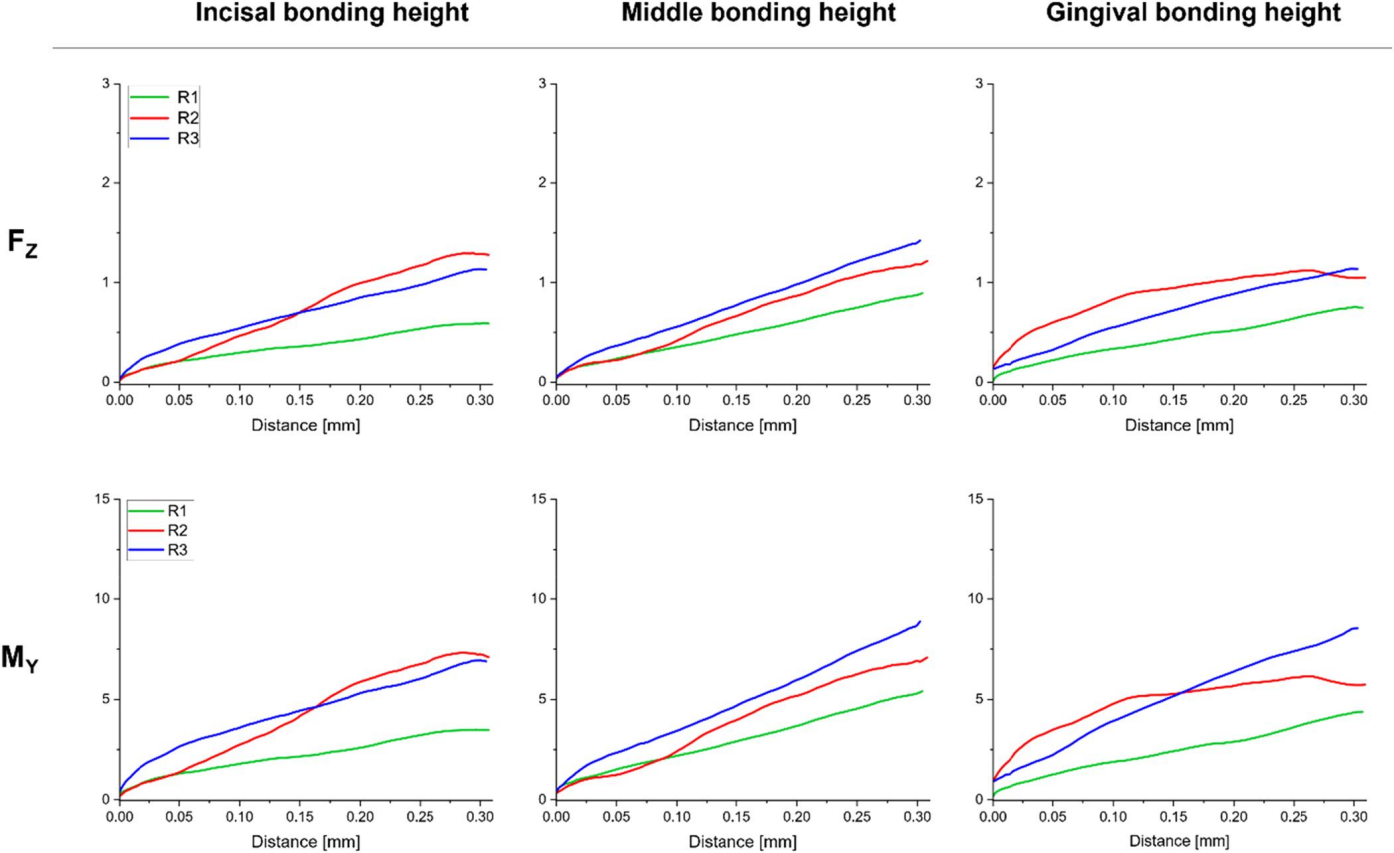
Diagrams depicting the development of the vertical force component *F*_Z_ (top) and of the labiolingual moment M_Y_ (bottom) in relation to an increasing vertical displacement for all included measurements.

Force and moment development curves with increasing vertical displacement for the different RBH are shown in Figure 4. The curves for R1 and R3 were similar in shape across the loading range, although they differed markedly in magnitude. R1 showed a steadily increasing response, whereas R2 displayed a less regular curve progression.

**Figure 5:**
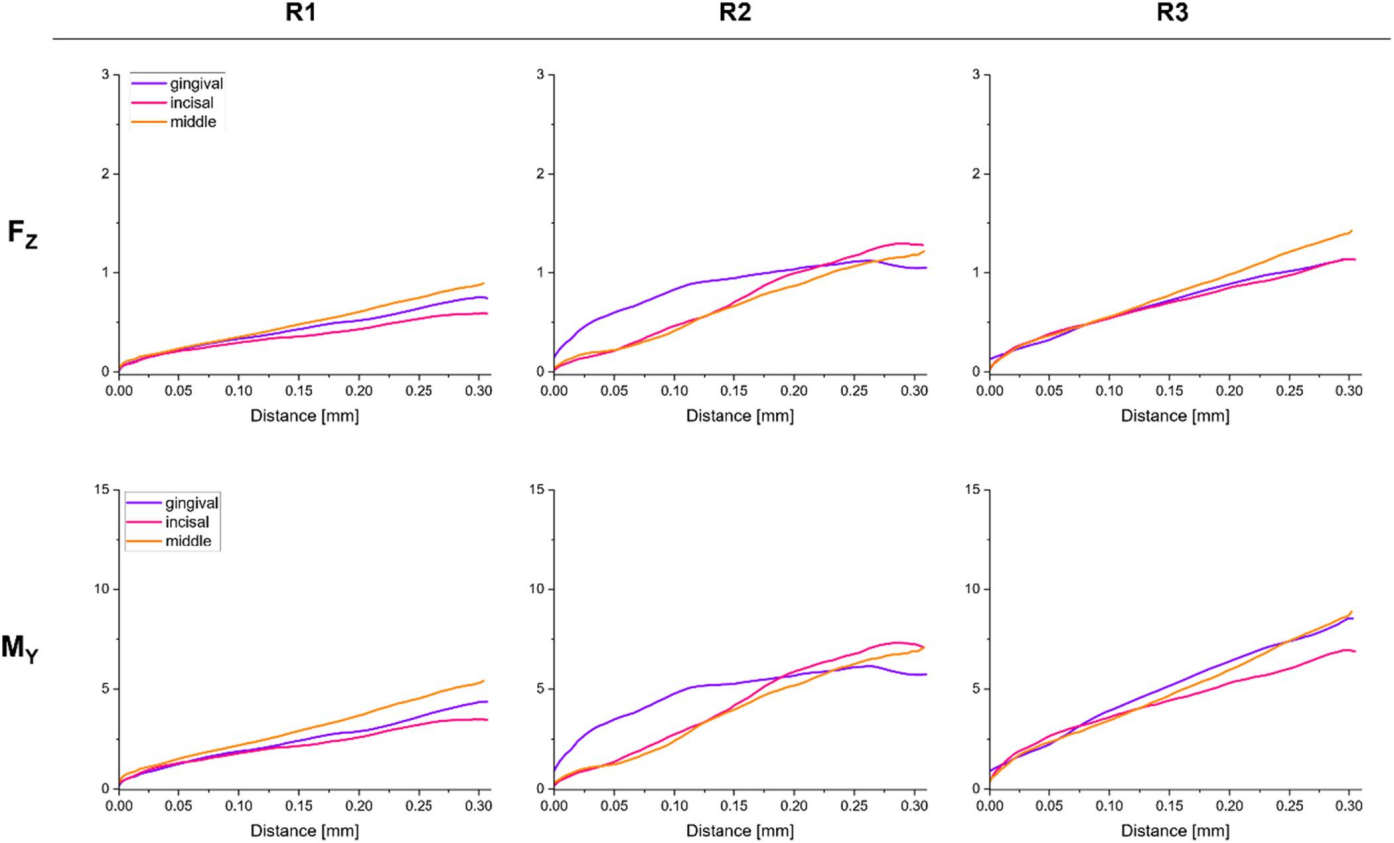
Line diagrams depicting the development of the vertical force component *F*_Z_ (top) and of the labiolingual moment *M*_Y_ (bottom) in relation to an increasing vertical displacement for all included measurements.

Force and moment development curves with increasing vertical displacement for the different retainer groups are shown in Figure 5.

Statistical analysis indicated that retainer type had a greater influence on force and moment development than RBH (Table 3). At the incisal RBH, *F*_Z_ differed significantly between R1 and R2 (*p* = 0.047), and between R1 and R3 (*p* = 0.006), but not between R2 and R3 (*p* = 1.000). For *M*_y,_ a significant difference was observed between R1 and R3 (*p* = 0.003). At the middle RBH, *F*_z_ and *M*_y_ did not differ significantly between groups. At the gingival RBH, statistically significant differences were observed between R1 and R2 (*F*_z_ *p* = 0.039; *M*_y_ *p* = 0.011), and R1–R3 (*F*_z_ *p* = 0.004; *M*_y_ *p* = 0.001).

**Table 3:** The statistical significance for vertical force (*F*_z_) and labiolingual moment (*M*_Y_) development for the entire measurement, in all possible comparisons between material groups.

RBH	Groups compared	*p* (*F*_Z_)	*p* (*M*_Y_)
Incisal	R1–R2	0.047*	0.078
	R2–R3	1.000	1.000
	R1–R3	0.006**	0.003**
Middle	R1–R2	1.000	1.000
	R2–R3	0.719	0.535
	R1–R3	0.057	0.055
Gingival	R1–R2	0.039*	0.011*
	R2–R3	1.000	1.000
	R1–R3	0.004**	0.001**

*Note:* The level of significance was *p* < 0.05. Significant results are denoted by * or **, based on the level of significance.

## 4 Discussion

RBH has been proposed as a biomechanical factor that may influence force‐moment generation in fixed lingual retainers and potentially contribute to adverse effects such as ITMs (Jahanbin et al. 2014; Padmos et al. 2018). Our data indicate that RBH at the incisal, middle, or gingival third of the crown did not significantly affect force or moment development upon vertical loading. In contrast, retainer type significantly influenced both outcomes, with R1 differing from the two other retainer types. Accordingly, the null hypothesis was partially rejected.

**Figure 6:**
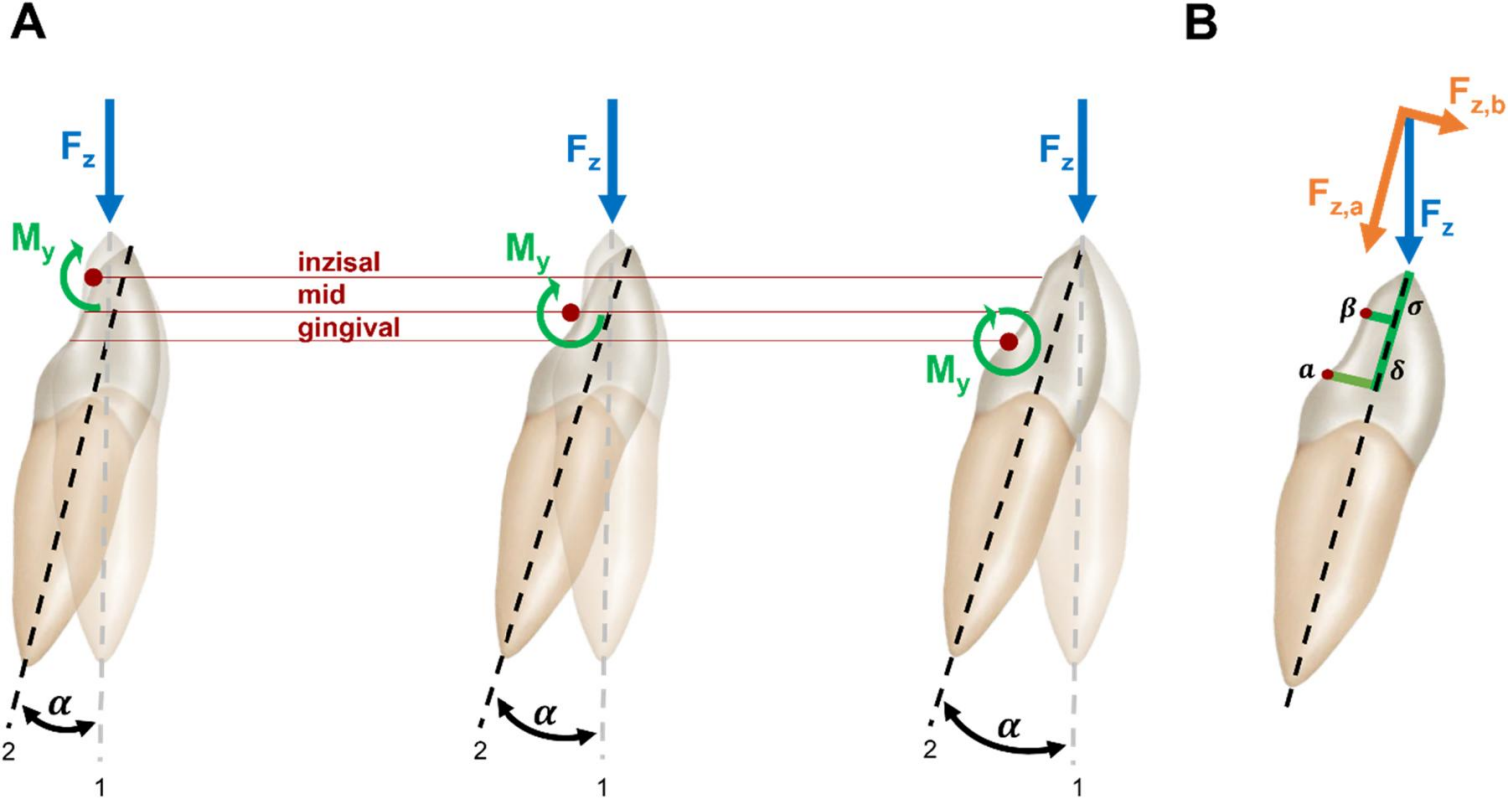
(A) Simplified representation of an axial vertical force (blue), which produces a labiolingual moment (green) acting at the center of rotation (red). The deflection angle *α*, which spans between the tooth's original position (1) and the new position that the tooth acquires due to an inadvertent tooth movement (2), is proportional to M through the lever r, and may be influenced by RBH. (B) Representation of a nonaxial vertical force *F*_Z_ (blue) acting on a lower canine, to which a retainer is bonded (red). The force is divided into two components, *F*_Za_ and *F*_Zb_ (yellow); *β* and *α* indicate the distance from the retainer to the tooth's axis. The lever *r*, or the distance between the point of force application and the retainer, is δ for an incisal RBH and δ + σ for a gingival RBH. The resulting moment for an incisally placed retainer is $M_{i}=F_{\mathrm{za}}\left(\beta \right)+\;F_{\mathrm{zb}}(\sigma )$, and, due to the longer lever *r*, it will be greater for a gingivally placed retainer: $M_{g}=\;F_{\mathrm{za}}\left(\alpha \right)+\;F_{\mathrm{zb}}(\delta +\sigma )$.

This was an unexpected outcome: since force (*F*) and moment (*M*) are connected via the lever (*r*), the distance between force application (i.e., the occlusal contact) and the retainer is theoretically directly proportional to the resulting labiolingual moment (*M*_Y_). When a vertical force (*F*_z_) is applied to the incisal edge, the retainer acts as the center of rotation (Figure 6A). A more gingivally bonded retainer, in which the lever *r* is greater, should present with an increase of *M*_Y_, or of angular deflection (*α*).

This should also be valid for nonaxial force application (Figure 6B), in cases with proclined or retroinclined incisors. However, this biomechanical concept alone cannot explain the findings of the present study, as no significant difference for force and moment development was observed between the analyzed RBHs. The highest forces and moments were measured at a middle RBH for the R1 and R3 group, and at an incisal RBH in the R2 group, suggesting that the response was not determined by bonding height alone but was also influenced by additional factors. These may include retainer type and material, stiffness, and the distance between the retainer and the point of load application, which may outweigh RBH‐related lever‐arm effects. Furthermore, possible variations in amount of composite resin, including thickness and coverage, can modify load transfer (Tee et al. 2023).

To date, only a few studies have investigated the biomechanical effects of RBH. Ohtonen et al. (2021) reported that greater forces were required to induce tooth displacement after axial loading when teeth were bonded at a more incisal RBH. However, although statistically significant, the observed difference was small, and displacement was assessed only for tooth 31 and limited to 0.1 mm. Similar conclusions were drawn in an in silico finite element method (FEM) study, in which a more incisal RBH was also associated with greater stability (Jahanbin et al. 2014). Notably, that analysis applied a comparatively high constant load of 10 N during displacement, and not all retainer types were evaluated across different RBHs.

The lower force and moment levels in the R1 group could be interpreted as low resistance of the retainer to loading, or as deformation of the wire. Previous research has suggested that round flexible wires, such as R1, could be more prone to “posttreatment activation” (Katsaros et al. 2007). Measurements obtained for the CAD/CAM fabricated retainer R3 were more homogeneous than those for R1 and R2, which suggests a more predictable response to loading. However, the evidence on CAD/CAM retainers is inconsistent. A recent clinical study reported comparatively high failure rates (Jowett et al. 2023), and an in vitro study found that conventional SS retainers outperformed the tested CAD/CAM retainers with respect to longevity and maximum load capacity (Roser 2023). At the same time, systematic reviews have not identified a clear clinical advantage or disadvantage for CAD/CAM retainers, largely due to limited and low‐certainty evidence (Martin 2023). In the present study, the CAD/CAM‐manufactured retainer R3 showed the fewest bonding failures and no retainer fractures.

The methodology of the present study was based on a previous investigation using the FRANS (Force/Torque Analysis System) test stand (Thaden et al. 2024), in which different retainer types and different loading scenarios were compared at a single bonding height. The present study investigated the effect of different RBHs, for which one representative loading scenario corresponding to incisor intrusion was selected. Future studies should investigate the effect of RBH at different incisor inclinations such as proclination or retroclination, since a varying inclination would shift the position of the force vector relative to the center of resistance and thereby alter the moment‐to‐force ratio. The retainer types were chosen to represent common manufacturing approaches (CAD/CAM, plier‐bent, hand‐bent). Since the R3 retainer was supplied with a silicone transfer guide, the same indirect bonding method was also selected for the two other retainers. However, to reflect clinical bonding conditions, no template was used to standardize the amount of composite. Consequently, the quantity of composite resin applied at each bonding site was not standardized, which may have affected the response of the retainers to loading. To limit the resulting variability, all retainers were bonded by a single trained operator following a standardized protocol, and repeated measurements were performed on identical model copies to account for the residual variance attributable to manual adhesive application. These copies were printed from a single STL master file on a high‐precision SLA 3D printer following a fixed protocol, with identical layer thickness, build orientation, and post‐processing to minimize variation between copies. The precision of resin‐based 3D‐printed orthodontic models has been reported as consistently below the 0.25 mm clinical threshold, with root‐mean‐square values of approximately 0.05 mm for SLA printers when the same master file is printed repeatedly (Grassia 2023). Nevertheless, a residual variation between copies cannot be entirely excluded.

As this was an in vitro study, the findings cannot be considered representative of in vivo behavior. The applied vertical loading protocol does not reproduce clinical functional loading, which is highly individual and occurs over extended time periods, often years. In the experiments, material fatigue was not accounted for, as each retainer was only loaded once, in contrast to dynamic and multidirectional masticatory forces. The experimental model also lacked a periodontal ligament (PDL) analog. Lastly, fixed retainers were only investigated bonded in the mandibular arch. Although no significant differences between RBHs were detected, height‐related effects may be more pronounced in the maxillary arch, where greater crown length increases the distance between RBHs.

When selecting retainer type and bonding height, clinicians should consider biomechanical aspects alongside oral‐hygiene and stability‐related factors. ITMs are most likely multifactorial in nature, with material‐related parameters playing a more dominant role than patient‐related factors in their development. Low force and moment expression, like those seen in the R1 retainers, could be interpreted as deformation of the wire due to loading, and, in consequence, as an expression of low resistance to loading. However, as this is an in vitro study, we cannot conclude that the use of an R1 wire would lead to ITM presentation in vivo. In the mandible, a more incisal RBH has been shown to be more advantageous clinically in the short term (Al‐Nimri and Al‐Qaqaa 2024), while in the maxilla, a gingival RBH is often necessary to avoid occlusal interferences. Our findings support bonding retainers at whichever bonding height is deemed to be the most appropriate in each individual situation. The higher levels of force and torque expressed in the R3 group could be interpreted as high resistance to loading, which would be beneficial for maintaining treatment stability. RBH has also been considered in clinical investigations pertaining to gingival and periodontal health (Tee et al. 2023; Jowett et al. 2023; Roser 2023), with some research finding no significant impact of RBH on periodontal outcomes (Jowett et al. 2023; Roser 2023), while a recent split‐mouth study (Tee et al. 2023) found significantly better gingival health parameters for the incisal RBH. In addition to this, recent clinical studies have shown that gingival and plaque indices were better in CAD/CAM retainers than in conventional twistflex wires (Knaup 2019; Bardideh 2023). In summary, this could imply that an incisally placed CAD/CAM retainer would be the ideal choice for periodontally healthy patients. Instead, in periodontally compromised patients, such as those with crestal resorption (Moradinejad 2025), more resilient retainers were recommended (Hetzler 2024). In this respect, the lower force and moment levels found in the round multistranded R1 may be the most suitable. Choosing an incisal bonding height would be advantageous as well, as it would aid in maintaining adequate oral hygiene (Al‐Nimri and Al‐Qaqaa 2024).

Since our findings did not determine significant differences between variations in RBH, clinical studies analyzing RBH in ITM cases would be of interest. In addition, as CAD/CAM‐fabricated retainers are increasingly being used in practice, further research is needed to characterize their biomechanical behavior.

## 5 Conclusion

RBH did not significantly affect the force and moment development during vertical loading in the biomechanical setup. In contrast, retainer type affected the development of forces and moments significantly in response to vertical loading. The significantly lower levels of forces and moment expression in multistranded round SS retainers may signify wire deformation and low resistance to torque.

## Author Contributions

**Francesca Thaden:** investigation, writing – original draft. **Linus Hötzel:** data curation, formal analysis, writing – review and editing. **Benedikt Dotzer:** visualization, software. **Matthias Mertmann:** conceptualization, methodology, writing – review and editing. **Andrea Wichelhaus:** supervision, resources, writing – review and editing. **Hisham Sabbagh:** methodology, writing – original draft, formal analysis, project administration.

## Funding

The authors have nothing to report.

## Ethics Statement

The authors have nothing to report.

## Consent

The authors have nothing to report.

## Conflicts of Interest

The authors declare no conflicts of interest.

## Permission to Reproduce Material From Other Sources

All content is original, and the authors hold all necessary rights.

## Data Availability

The data that support the findings of this study are available from the corresponding author upon reasonable request.
